# Pod-Like Supramicelles with Multicompartment Hydrophobic Cores Prepared by Self-Assembly of Modified Chitosan

**DOI:** 10.1007/s40820-015-0070-4

**Published:** 2015-10-27

**Authors:** Yiming Wang, Jie Wang, Tongshuai Wang, Yisheng Xu, Lei Shi, Yongtao Wu, Li Li, Xuhong Guo

**Affiliations:** 1 State Key Laboratory of Chemical Engineering, East China University of Science and Technology, Shanghai, 200237 People’s Republic of China; 2 Firmenich Aromatics (China) Co., Ltd., Shanghai, 201108 People’s Republic of China

**Keywords:** Chitosan, Graft copolymers, Amphiphiles, Self-assembly, Multicompartment supramicelles

## Abstract

In this paper, pod-like supramicelles with multicompartment hydrophobic cores were prepared by self-assembly of amphiphilic *N*-phthaloylchitosan-g-poly(*N*-vinylcaprolactam) (PHCS-g-PNVCL) in aqueous medium. The employed biocompatible amphiphilic polymer was synthesized by grafting the carboxyl terminated poly(*N*-vinylcaprolactam) (PNVCL-COOH) chains onto *N*-phthaloylchitosan (PHCS) backbone. ^1^H NMR and FTIR results confirmed the molecular structure of the copolymers. The morphology of the supramicelles assembled by PHCS-g-PNVCL was revealed by means of TEM and polarized light microscope. In solution, the supramicelles were very stable as monitored by DLS and zeta potential measurements. Temperature and pH presented significant influences on the size and size distribution of the supramicelles. These supramicelles with multicompartment hydrophobic cores should be ideal biomimetic systems with promising applications in drug delivery.

**Electronic supplementary material:**

The online version of this article (doi:10.1007/s40820-015-0070-4) contains supplementary material, which is available to authorized users.

## Introduction

In nature, compartmentalization is one of the essential requirements for life. For example, the cell membrane has the ability to keep intracellular components together and protect them from an outside environment. This nature’s ability to achieve multiple levels of compartmentalization has attracted scientists’ attention and motivated them to explore how compartmentalization can be established by artificial materials [1]. Multicompartment micelles, with a hydrophilic corona and a microphase-separated hydrophobic core, have received increasing attention over the last decade [2–5]. Due to their intrinsic properties, multicompartment micelles can selectively entrap and release different hydrophobic compounds simultaneously [3], thus are promising for a wide range of applications especially for drug delivery [2, 6]. Currently, the main strategy to design and prepare multicompartment micelles is through self-assembling some block copolymers with a water-soluble segment and two or more mutually incompatible hydrophobic segments, such as hydrocarbon and fluorocarbon [7–11]. According to previous theoretical study, amphiphilic graft copolymers should also be able to assemble into multicompartment micelles as long as they have hydrophobic chains with sufficient incompatibility [12]. However, to the best of our knowledge, few graft copolymer-based multicompartment micelles have been reported.

Chitosan is a natural alkaline polysaccharide extracted from the shell of crustaceans and is composed of randomly distributed β-(1, 4)-linked d-glucosamine and *N*-acetyl-d-glucosamine units [13]. Because of its intrinsic properties, such as non-toxic, biocompatible, and biodegradable [14–16], chitosan has been widely utilized in biological and pharmaceutical field, such as tissue engineering [17, 18], wound healing [19, 20], bioimaging [21, 22], and drug delivery [23, 24].

In this study, chitosan-based pod-like supramicelles with multicompartment hydrophobic cores were formed by assembly of chitosan-based amphiphilic graft copolymers. These novel biomaterial-based supramicelles combined the specifics of multicores and multicompartments simultaneously. At first, densely phthalic anhydride-grafted *N*-phthaloylchitosan (PHCS) was obtained by introducing phthalic anhydride into the backbone of chitosan. Subsequently, biocompatible monocarboxyl-terminated poly(*N*-vinylcaprolactam) (PNVCL-COOH) was grafted onto the backbone of PHCS by coupling reaction to give the terminal amphiphilic graft copolymer PHCS-g-PNVCL. All the intermediates and product were confirmed by proton nuclear magnetic resonance (^1^H NMR) and Fourier transform infrared spectroscopy (FTIR). The critical aggregation concentration (CAC) was determined by fluorescent spectroscopy using pyrene as fluorescent probe. The morphology of the prepared supramicelles was investigated by transmission electron microscopy (TEM) and polarized light microscope (PLM). Dynamic light scattering (DLS) was employed to study the stability of the supramicelles in 5 months.

## Experimental Section

The detailed procedure on the synthesis of the graft copolymer, preparation of supramicelles, and all characterization techniques are available in the supplementary information.

## Results and Discussion

**Fig. 1:**
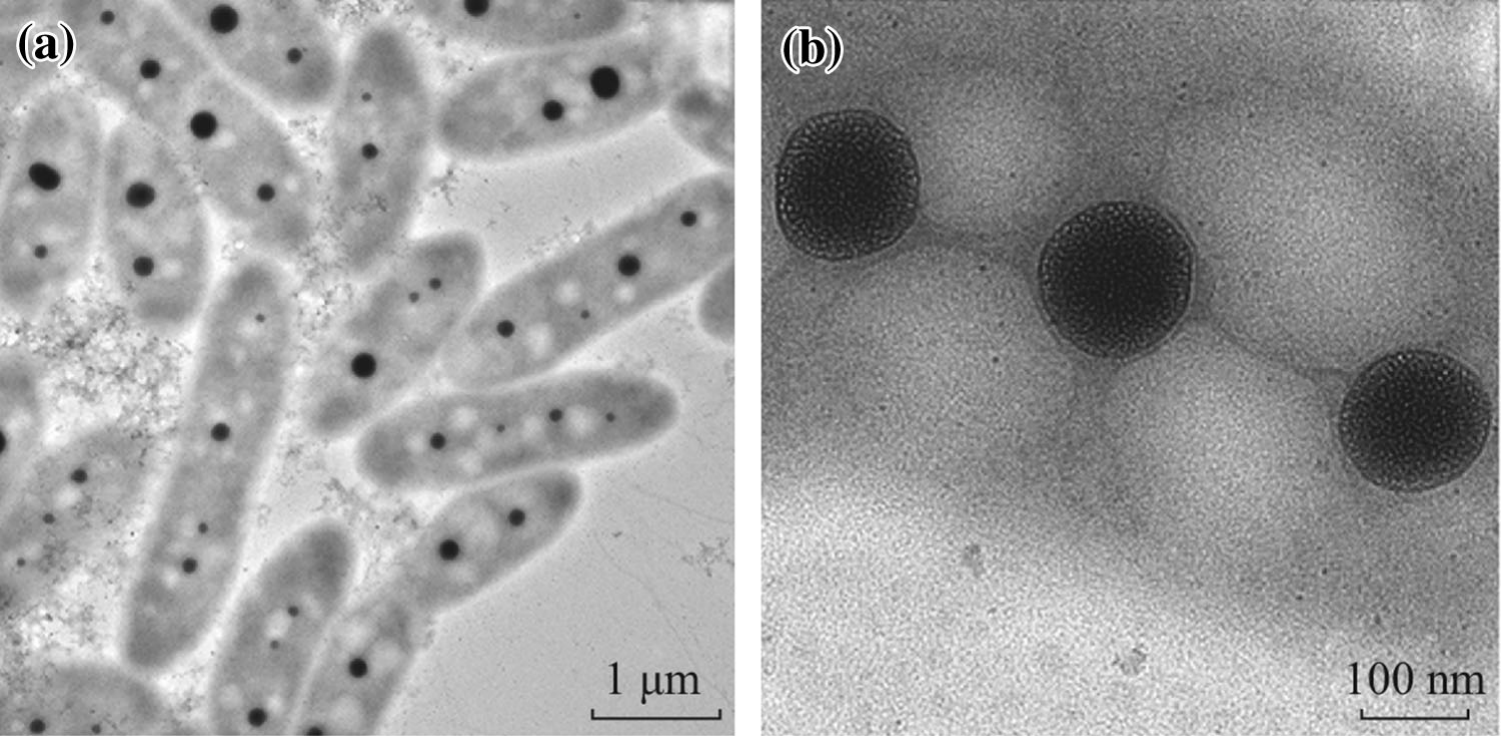
TEM images of the supramicelles: the pod-like supramicelles (**a**), and the intrastructure of the pod-like supramicelle (**b**)

**Fig. 2:**
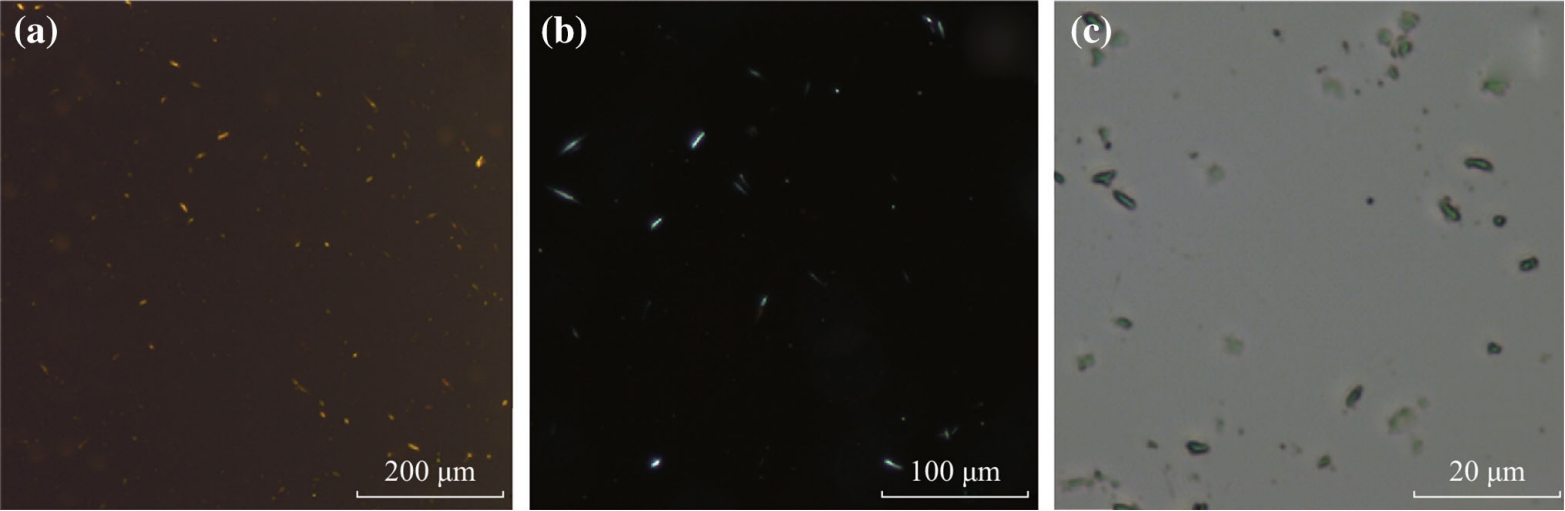
PLM images of the supramicelles under different magnifications. The *scale bars* are: **a** 200 μm (excitation light, *λ*_ex_ = 440–460 nm), **b** 100 μm (excitation light, *λ*_ex_ = 390–410 nm), and **c** 20 μm (natural light)

With an amphiphilic graft copolymer (PHCS-g-PNVCL) of hydrophobic groups and hydrophilic polymer chains, a novel supramicelle with several multicompartment hydrophobic cores in pod-like shape was prepared by the typical precipitation method [25]. As shown by TEM in Fig. 1a and b, the synthesized graft copolymer PHCS-g-PNVCL self-assembled into pod-like supramicelles [about (2.5 ± 1) × (0.8 ± 0.2) μm^2^] with several hydrophobic cores (around 180 nm) and a supra hydrophilic corona. The PLM images (Fig. 2) provided an original figure of the assembled structure. Although the internal structure of the aggregates could not be visualized by PLM, the shape of the aggregates could be clearly identified as pod-like consistent with the TEM result.

Figure 1b shows that these pod-like supramicelles are consisted of several mono hydrophobic cores and a supra corona. It could be seen clearly that these mono hydrophobic cores are formed by light gray regions as well as darker domains. These light gray regions and darker domains are regarded as the formation of hydrophobic compartments [5]. The formation of these mini compartments could be attributed to the intramolecular self-assembly of the hydrophobic phthalic anhydride groups. Under aqueous condition, these phthalic anhydride groups inclined to aggregate spontaneously into mini hydrophobic patches. The size of these mini patches was determined to be 4 ± 0.5 nm by TEM. These mini-domains distributed homogeneously in the hydrophobic cores which might be attributed to the regular arrangement of the phthalic anhydride-modified chitosan [26]. These continuous and homogeneous distributed domains provided a great deal of mini-hydrophobic compartments in the hydrophobic cores which presented promising applications in pharmaceutical field, such as drug delivery.

**Fig. 3:**
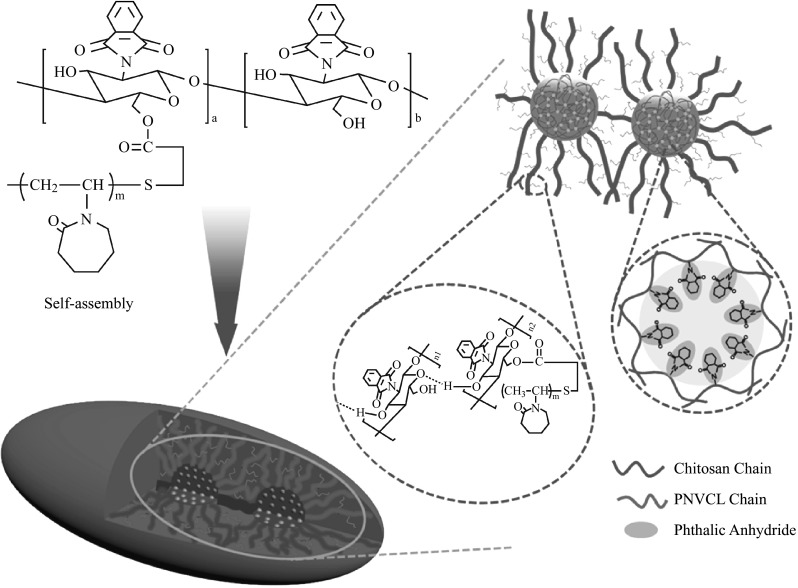
Chemical structure of the PHCS-g-PNVCL copolymer and the hypothetical self-assembly mechanism for the formation of the pod-like supramicelles

The self-assembly was driven by the hydrophobic interactions between the grafted phthalic anhydride groups and the hydrogen bonding between chitosan chains. Figure 3 shows the hypothetical mechanism of the self-assembly procedure. Amphiphilic copolymers are able to self-assemble into aggregates with complex and well-defined morphology under appropriate conditions. The self-assembly behavior can be determined not only by the hydrophilic/hydrophobic balance of the intramolecular but also by the copolymer topology [27]. For example, the amphiphilic copolymers with densely grafted hydrophobic groups could collapse and curl to form the micelles with multi-hydrophobic cores which are vividly called pearl necklace micelles in aqueous medium [28–30]. In this work, the densely grafted hydrophobic phthalic anhydride groups of PHCS-g-PNVCL rendered the collapse of the copolymer main chains, intermolecular and intramolecular aggregates formed subsequently.

It is hypothesized that the main chains of PHCS-g-PNVCL collapsed and curled initially to form a spherical hydrophobic core under aqueous condition. As the TEM image shown in Fig. S4A, it could be seen clearly that a lot of cores (black domains) generated at the initial time. And then, the rest of these main chains after the intramolecular assembly changed their spatial configuration spontaneously to form apparent hydrophilic chains in water under the hydrophobic interactions and hydrogen bonding. These apparent hydrophilic chains and the grafted hydrophilic PNVCL chains stretched into aqueous medium to form a hydrophilic corona to stabilize the micelles (as the amplified graph shown in Fig. 3). Because of the hydrogen bonding and the hydrophobic interactions between the main chains of the copolymers, several mono micelles would link together to generate the reported pod-like supramicelles with several hydrophobic cores and a supra hydrophilic corona, as shown in Fig. 3. It could also be observed from the TEM images in Fig. S4B-C that the polymers assembled into a micelle with a tail (aggregates of the rest of the main chains) which might be a transit state in the formation procedure of the supramicelles, and then these mono micelles were linked together by their tails to form the pod-like supramicelles, as shown in Fig. S4D.

Figure 3 also delineates the internal structure of the mini-micelles in the hydrophobic core. From this hypothesized schematic diagram, the PHCS chains curled together and the grafted hydrophobic phthalic anhydride groups assembled into hydrophobic mini-cores to reach a stable state in aqueous medium.

The CAC of the synthesized amphiphilic copolymer was determined by fluorescence spectroscopy using pyrene as fluorescent probe [31]. Among the five peaks in the emission spectra of pyrene, the intensity ratio of the first peak at 372 nm and the third peak at 385 nm (I372/I385) is very sensitive to the polarity of the micro-aqueous medium [32]. As shown in Fig. S3, a low CAC value of 0.46 mg l^−1^ was determined for PHCS-g-PNVCL. This low CAC value indicated that PHCS-g-PNVCL could be applicable under highly diluted conditions.

**Fig. 4:**
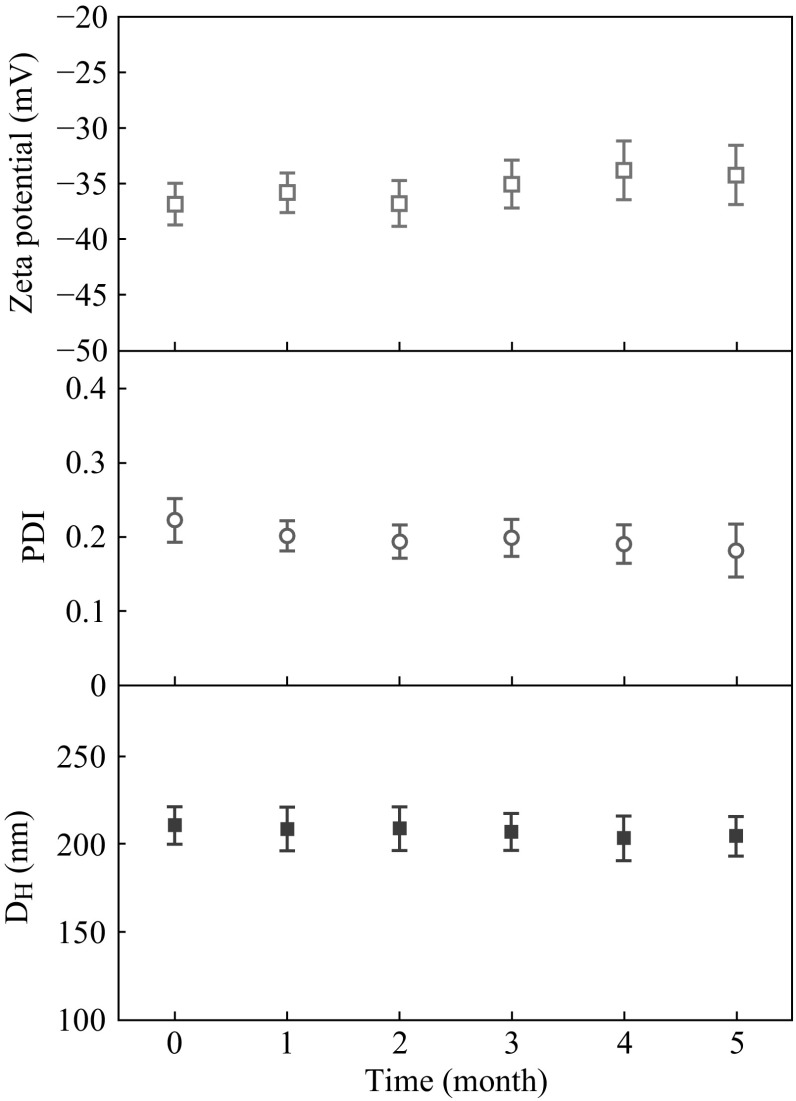
The size (*filled square*), PDI (*open circle*), and zeta potential (*open square*) of the supramicelles recorded by DLS in 5 months

To evaluate the stability of the supramicelles, the hydrodynamic properties of this colloidal system were monitored by DLS equipped with a Malvern Zetasizer Nano ZS instrument. It is noted that hydrodynamic size given by DLS could be employed to characterize the stability of these supramicelles, although DLS gives a spherical-averaged size that is equal to neither the pod length nor the pod width [33]. As shown in Fig. 4, the size and PDI of the supramicelles had no obvious change during 5 months. Another important colloid property, zeta potential, [34], also remained constant at −34 ± 2 mV, which also demonstrated the excellent stability of the prepared supramicelles. This remarkable stability might be attributed to the stiff hydrophobic cores confined by hydrogen bonding, hydrophobic interactions, and the steric hindrance from the hydrophilic corona. The stability investigation indicated that this prepared supramicelles could be the promising candidates for drug delivery applications.

Furthermore, we found that pH and environmental temperature presented profound effects on the assembly of these supramicelles. TEM was carried out to observe the morphology of the supramicelles assembled at different pH (Fig. S5). Correspondingly, size and size distribution of these supramicelles were monitored by DLS (Fig. S6). The size of the supramicelle increased significantly as the pH stood at an acid level, and the size distribution was very broad which had been reached above 1.0, as shown in Fig. S6. An intuitional observation given by TEM showed that most of the supramicelles aggregated together with a disordered shape. We could reasonably assume that the protonation destroyed the hydrogen bonding and the hydrophilic/hydrophobic balance. However, the supramicelles became more uniform while the pH increased at a higher level (above 7.0), the size presented a negligible variation, but the PDI increased slightly when the pH was increased above 8.0, which might be attributed to the high ionic strength. Temperature was also an important factor which could influence the size and size distribution of the supramicelles. As shown in Fig. S7, after a slight increase before reaching at 32 °C, the size of the supramicelles increased dramatically as the temperature increased from 32 to 38 °C, and reached at an equilibrium level after 40 °C. This might be attributed to the thermo-sensitive grafting polymer PNVCL. When the temperature increased above its lower critical solution temperature (about 32 °C), the PNVCL under hydrophobic state would like to stack onto the surfaces of the hydrophobic cores, as a result, the size of the supramicelles increased. While the temperature increased above 38 °C, most of the PNVCL chains had been translated into hydrophobic state, correspondingly, the size would reach at a constant value. These investigations indicated that pH and temperature are two important factors which could be employed to control the size and size distribution of the reported supramicelles.

## Conclusions

In this study, a novel amphiphilic graft copolymer PHCS-g-PNVCL was synthesized by a “graft onto” method. An interesting pod-like supramicelle with several multicompartment hydrophobic cores and a supra corona was prepared in aqueous medium. The hydrogen bonding and hydrophobic interactions between the PHCS chains are supposed to be the main driving force of the self-assembly. The supramicelles had a pod-like shape with a size of (2.5 ± 1) × (0.8 ± 0.2) μm^2^ and the hydrophobic cores were around 180 nm. The supramicelles demonstrated excellent stability in aqueous medium for 5 months. The pH value and temperature are two factors to influence the size and size distribution of the supramicelles. These novel pod-like supramicelles with multicompartment hydrophobic cores provide a promising microscale structure for a wide range of applications such as templating materials, solubilization, and drug delivery.

## Electronic supplementary material

Below is the link to the electronic supplementary material.

Supplementary material 1 (PDF 880 kb)
