# Supplementary material for: Pod-Like Supramicelles with Multicompartment Hydrophobic Cores Prepared by Self-Assembly of Modified Chitosan
Source: Nanomicro Lett. 2015 Oct 27;8(2):151–6. doi: 10.1007/s40820-015-0070-4 (PMC6223668; doi:10.1007/s40820-015-0070-4)
Supplement: Supplementary file 1 — Supplementary material 1 (PDF 880 kb) [file 40820_2015_70_MOESM1_ESM.pdf]

Supplementary Information for

Pod-like Supramicelles with Multicompartment Hydrophobic Cores Prepared by Self-assembly of Modified Chitosan

Yiming Wang[1], Jie Wang[1,*], Tongshuai Wang[1], Yisheng Xu[1], Lei Shi[2], Yongtao Wu[2], Li Li[1], Xuhong Guo[1,*]

[1]State Key Laboratory of Chemical Engineering, East China University of Science and Technology, Shanghai 200237, People's Republic of China

[2]Firmenich Aromatics (China) Co., Ltd., Shanghai 201108, People's Republic of China

*Corresponding authors. E-mail: jiewang2010@ecust.edu.cn, guoxuhong@ecust.edu.cn

1 Materials

Chitosan (CS, degree of *N*-deacetylation=95 %, Mw = 50 kDa) was purchased from Aoxing Biotechnology Co. Ltd., China, and used as received. Phthalic anhydride was supplied by the First Reagent Factory of Shanghai, China. *N*-Vinylcaprolactam (NVCL), 2, 2'-azoisobutyronitrile (AIBN), 3-mercaptopropionic acid (MPA) and 1-ethyl-3-(3-dimethylaminopropyl) carbodiimide (EDC) were obtained from J&K Scientific Ltd., China, and NVCL was recrystallized from hexane for three times before being used. 1-Hydroxybenzotrizole (HOBt), 1,4-Dioxane and *N*, *N*-dimethylformamide (DMF) were purchased from Sinopharm Chemical Reagent Co., Ltd. (Shanghai, China), and distilled under reduced pressure from calcium

hydride. The water used in all experiments was purified by reverse osmosis (Shanghai RO Micro Q). All other reagents and solvents were analytical grade and used without further purification.

2 Synthesis and Methods

2.1 Synthesis of PHCS-g-PNVCL Copolymer

Synthesis of PHCS: Chitosan (5 g, 31.01 mmol pyranose units) and phthalic anhydride (13.8 g, 93.03 mmol) were dissolved in DMF (150 mL) and reacted at 125 °C under the protection of nitrogen atmosphere for 5 h. The product was precipitated in cold water and washed with methanol for 3 times to give 8.16 g pale yellow products after vacuum drying. The degree of substitution (DS) of phthaloyl groups within PHCS was determined to be about 1.12 by elemental analysis (EA) and the yield of PHCS was about 80.5 % based on the data of EA (Table S1).

Synthesis of PNVCL-COOH: NVCL (2.5 g, 17.96 mmol), MPA (0.24 g, 2.26 mmol), and AIBN (0.05 g, 0.30 mmol) were dissolved in 20 mL purified 1, 4-Dioxane. The solution was degassed by flushing with nitrogen for 30 min. The polymerization was subsequently conducted under stirring at 68 °C for 24 h. After the reaction, the product was precipitated with excess amount of diethyl ether and dried under vacuum at 45 °C overnight to obtain 2.03 g (yield 81.2 %) white powder.

Synthesis of PHCS-g-PNVCL copolymer: PHCS (0.30 g, about 1.08 mmol phthaloylated pyranose units) was stirred with PNVCL-COOH (1.2 g, 1.08 mmol) in

purified DMF solution. Then HOBt (0.44 g, 3.24 mmol) and EDC (0.64 g, 3.24 mmol) were added into the mixed solution and reacted at room temperature for 48 h. The obtained mixture was dialyzed against distilled water and washed with excess ethanol to obtain 0.324 g white powder. The graft content (GC) was 8.0 %, calculated as follows:

$$GC(\%) = \frac{m_g - m_o}{m_o} \times 100 \qquad (1)$$

where m_g and m_o are the weight of PHCS-g-PNVCL and PHCS, respectively.

2.2 Preparation of PHCS-g-PNVCL Based Pod-like Supramicelles

5 mg PHCS-g-PNVCL was dissolved in 2 mL DMF and then added drop-wise into distilled water under vigorous stirring. The obtained colloid solution was transferred into a dialysis bag (MWCO=8-12 kDa) and dialyzed against distilled water for 24 h at an interval of 6 h to give the supramicelles.

3 Characterizations

All infrared spectra were obtained from samples in KBr pellets using a Nicolet 6700 FT-IR spectrophotometer. ^{1}H NMR spectra were measured on a 500 MHz Bruker DRX500 spectrometer at 25 °C. Elemental analysis was performed using a VARIO EL III elemental analyzer. Critical aggregation concentration (CAC) was determined using a RF-540 spectrophotofluorimeter of SHIMADZU with pyrene as the probe (excited at 335 nm). The hydrodynamic properties were determined by dynamic light

scattering (DLS) (Particle Sizer NICOMP 380 ZLS instrument) at a scattering angle of 90 °. TEM was performed a JEM-2100 electron microscope by operating at 200 kV. Morphology of the prepared supramicelles was also observed by using a LEICA DM2500P polarized light microscope (PLM) equipped with a fluorescence module (the samples were observed under both normal light and excitation light). Images were captured using a CCD camera connected to a PC via a WT-1000GM imaging board.

4 Supporting Results and Discussion

4.1 Synthesis and Characterization of the PHCS-g-PNVCL Copolymer

In Fig. S1, the ^{1}H NMR spectrum for PNVCL-COOH showed peaks at 4.2 ppm (1H, -NCH-), 3.2 ppm (2H, $-NCH_2-$), 2.3 ppm (2H, $-COCH_2-$), and 1.1-1.9 ppm (6H, $-CH_2-$ of the caprolactam ring, and 2H, $-CH_2-$ of the PNVCL backbone). Additionally, two peaks at 2.8 and 2.95 ppm which were attributed to MPA. In the FT-IR spectrum of PNVCL-COOH (Fig. S2), the strong peaks at 1631 and 1480 cm^{-1} were attributed to the amide I band and C-N stretching vibration, while the peaks of monomer NVCL at 1658 cm^{-1} (C=C) and 3000-3100 cm^{-1} (C=C and CH=) disappeared. The analysis above indicated that the PNVCL-COOH was synthesized successfully. The molecular weight of PNVCL-COOH ($M_{\text{PNVCL-COOH}}$) was determined to be 1100 Da by ^{1}H NMR according to the following method:

$$M_{PNVCL-COOH} = \frac{2\times I_{4.2\,ppm}}{I_{2.8\,ppm}} \times M_{NVCL} \qquad (2)$$

where $I_{4.2ppm}$ and $I_{2.8ppm}$ were the signal intensities for -NCH- of PNVCL-COOH and the -CH_2- of the terminal MPA group, M_{NVCL} was the molecular weight of NVCL monomer.

In the ^{1}H NMR spectrum of PHCS (Fig. S1), two broad peaks, 2.8-5.0 ppm and 7.8-8.0 ppm, corresponding to the hydrogen of chitosan backbone and phthaloyl groups were observed. As shown in the FT-IR spectrum of PHCS (Fig. S2), the characteristic peaks at 1776.1 and 1712.5 cm^{-1} were attributed to the carbonyl anhydride and the peak at 721.3 cm^{-1} was referred to the aromatic ring. These results confirmed the molecular structure of PHCS.

In the ^{1}H NMR spectra (Fig. S1), PHCS-g-PNVCL presented new proton peaks at 1.1-1.9 ppm and 3.1-3.2 ppm, which were attributed to the caprolactam ring and the -SCH_2CH_2- group, respectively. Compared to PHCS, the FTIR spectrum of PHCS-g-PNVCL (Fig. S2) showed new absorption peaks at 1186.2 and 1089.5 cm^{-1}, which belonged to the dissymmetrical and symmetry stretching vibration peaks of the ester linker, respectively. The absorbance at 1634.5 and 1643.5 cm^{-1} were attributed to the carbonyl and -CH_2CH_2- group, respectively. The ^{1}H NMR and FT-IR analysis of PHCS-g-PNVCL indicated that the PNVCL-COOH chains were successfully grafted onto the PHCS backbone.

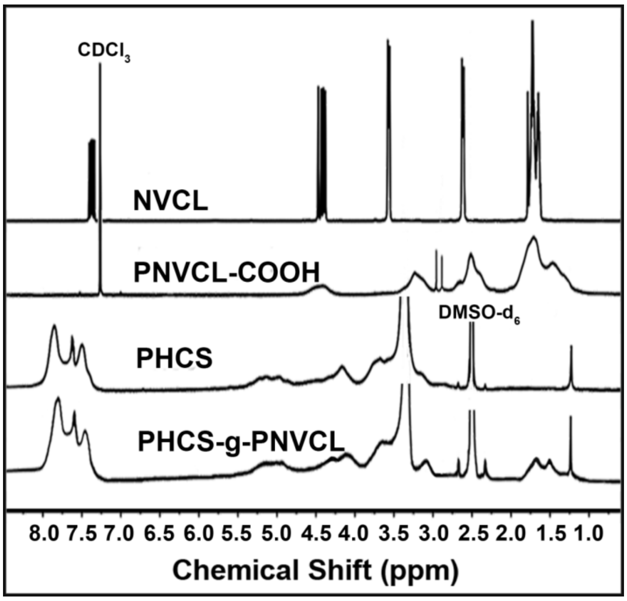

Fig. S1 ^{1}H NMR spectra of NVCL monomer, PNVCL-COOH, PHCS, and PHCS-g-PNVCL

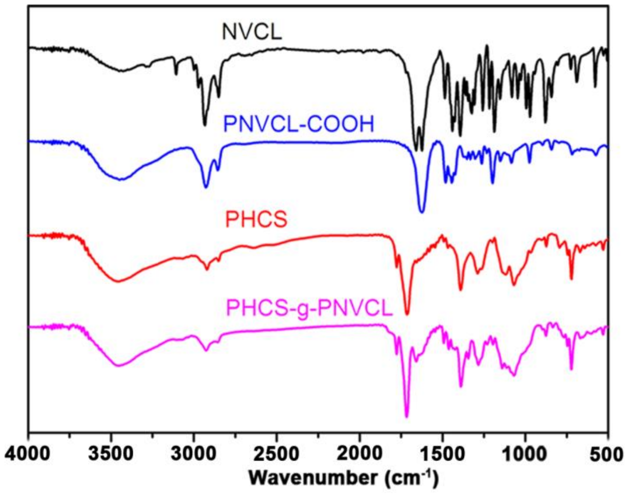

Fig. S2 FT-IR spectra of NVCL, PNVCL-COOH, PHCS, and PHCS-g-PNVCL

4.2 Elemental Analysis

Table S1 The elemental analysis results of PHCS.

PHCS (mg)	(*C*) wt%	(*N*) wt%	(*H*) wt%
2.291	55.73	4.58	4.88

According to the ^{1}H NMR spectra (Fig. S1), we can reasonably assume the molecular composition of PHCS as: $(C_{22}H_{17}NO_9)_x.(C_{14}H_{13}NO_6)_y.$ z•H_2O. Then the values of x, y, and z can be calculated with the following equations:

$$x+y=1 \quad (3)$$

$$\frac{264x+168y}{M}=(C)wt\% \quad (4)$$

$$\frac{14x+14y}{M}=(N)wt\% \quad (5)$$

$$\frac{17x+13y+2z}{M}=(H)wt\% \quad (6)$$

Calculated results: $x=0.88$, $y=0.12$ and $z=0.72$

Therefore, the molecular composition of the synthesized PHCS could be expressed as: $(C_{14}H_{13}NO_6)_{0.88}(C_{22}H_{17}NO_9)_{0.12}$ 0.72H_2O (C, 55.73 %; N, 4.58 %; H, 4.88 %). This calculation results could match theory formula. Thus the DS of phthaloyl groups within PHCS could be determined to be 1.12.

4.3 Amphiphilic Property of PHCS-g-PNVCL

The amphiphilic property of PHCS-g-PNVCL was investigated by fluorescence method using pyrene as the fluorescence probe. The aggregated pyrene would emit strong fluorescence. Among the five peaks emitted by the aggregated pyrene, the intensity ratio of the first peak at 372 nm and the third peak at 385 nm (I_{372}/I_{385}) is very sensitive to the polarity of the micro aqueous media [1]. As shown in Fig. S3, when the concentration of PHCS-g-PNVCL was lower than the CAC, the copolymer dispersed in the aqueous medium as single chains and the I_{372}/I_{385} ratio kept in constant. The ratio decreased dramatically when the concentration increased above the

CAC. This result was attributed to the encapsulation of pyrene by the formed micelles. The CMC of PHCS-g-PNVCL was determined to be 0.46 mg L^{-1}.

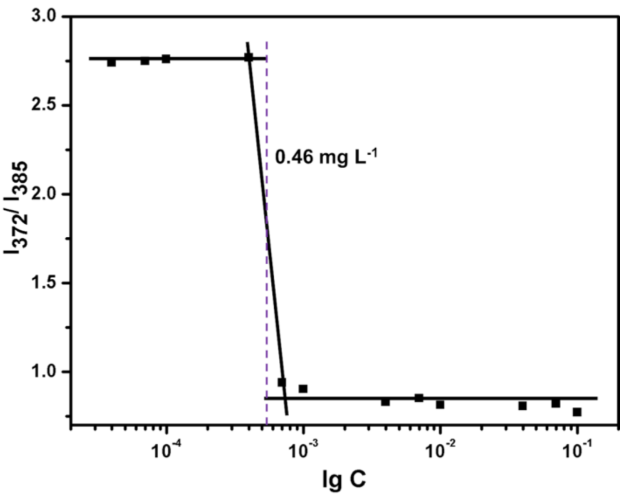

Fig. S3 Plot of the intensity ratio (I_{372}/I_{385}) in the pyrene fluorescence spectrum as a function of PHCS-g-PNVCL concentration. λ_{ex} = 335 nm. [pyrene] = 6.0×10^{-7} M.

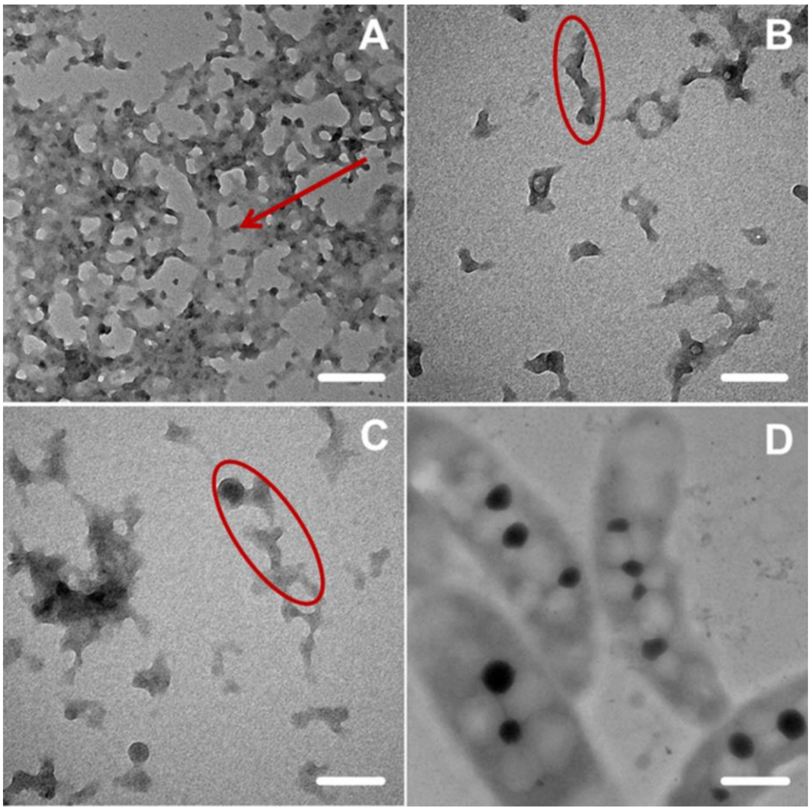

Fig. S4 The morphology of the supramicelles at different time determined by TEM: (A) 1 h, (B) 6 h, (C) 12 h, and (D) 24 h after dropping the preparation of the supramicelles (Scale bar = 200 nm)

4.4 Influences of pH and Temperature on the Morphology of the Supramicelles

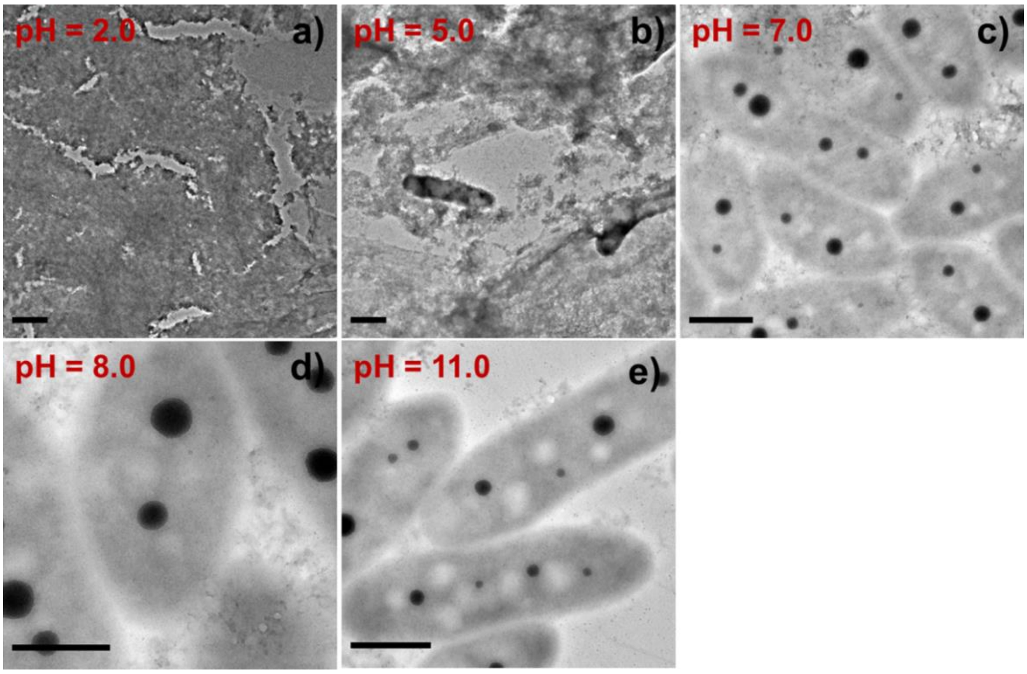

Fig. S5 TEM images of the supramicelles under different pH conditions: a) pH=2.0; b) pH=5.0; c) pH=7.0; d) pH=8.0; e) pH=11.0 (scale bar = 1μm)

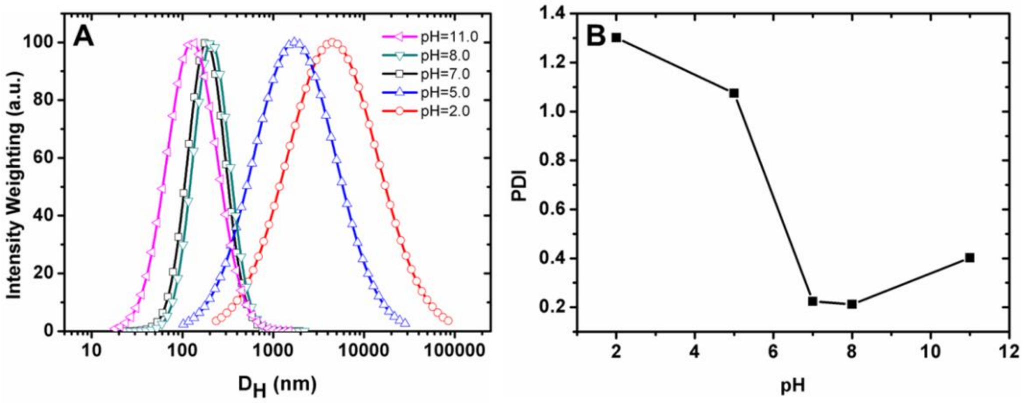

Fig. S6 Size (A) and Size distribution (B) of the supramicelles under the conditions with different pH given by DLS

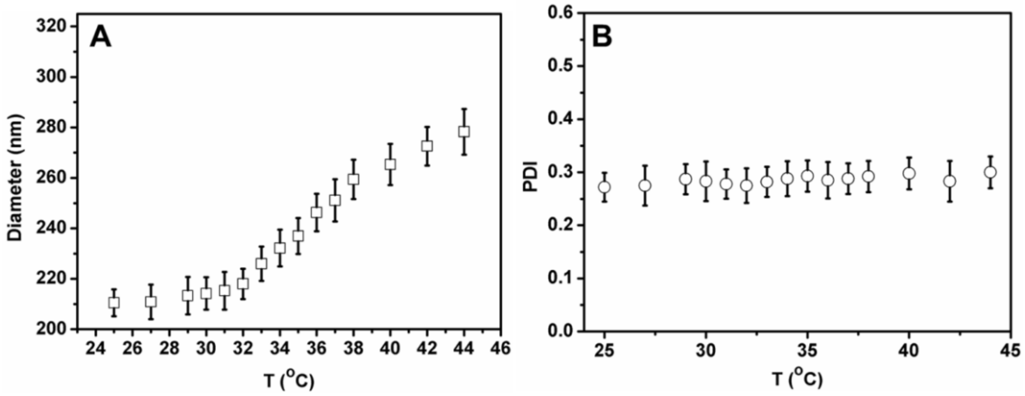

Fig. S7 Size (A) and size distribution (B) of the supramicelles at different temperature given by DLS

Reference

[1] K. Kalyanasundaram, J.K. Thomas, Environmental effects on vibronic band intensities in pyrene monomer fluorescence and their application in studies of micellar systems. JACS **99**(7), 2039–2044 (1977). doi:10.1021/ja00449a004
